# Towards a Pharmacophore for Amyloid

**DOI:** 10.1371/journal.pbio.1001080

**Published:** 2011-06-14

**Authors:** Meytal Landau, Michael R. Sawaya, Kym F. Faull, Arthur Laganowsky, Lin Jiang, Stuart A. Sievers, Jie Liu, Jorge R. Barrio, David Eisenberg

**Affiliations:** 1 Howard Hughes Medical Institute, UCLA-DOE Institute for Genomics and Proteomics, Departments of Biological Chemistry and Chemistry and Biochemistry, University of California, Los Angeles, California, United States of America; 2 The Pasarow Mass Spectrometry Laboratory, The NPI-Semel Institute for Neuroscience and Human Behavior, David Geffen School of Medicine, University of California, Los Angeles, California, United States of America; 3 Department of Molecular and Medical Pharmacology, David Geffen School of Medicine, University of California, Los Angeles, California, United States of America; University of California San Francisco/Howard Hughes Medical Institute, United States of America

## Abstract

Diagnosing and treating Alzheimer's and other diseases associated with amyloid fibers remains a great challenge despite intensive research. To aid in this effort, we present atomic structures of fiber-forming segments of proteins involved in Alzheimer's disease in complex with small molecule binders, determined by X-ray microcrystallography. The fiber-like complexes consist of pairs of β-sheets, with small molecules binding between the sheets, roughly parallel to the fiber axis. The structures suggest that apolar molecules drift along the fiber, consistent with the observation of nonspecific binding to a variety of amyloid proteins. In contrast, negatively charged orange-G binds specifically to lysine side chains of adjacent sheets. These structures provide molecular frameworks for the design of diagnostics and drugs for protein aggregation diseases.

## Author Summary

The devastating and incurable dementia known as Alzheimer's disease affects the thinking, memory, and behavior of dozens of millions of people worldwide. Although amyloid fibers and oligomers of two proteins, tau and amyloid-β, have been identified in association with this disease, the development of diagnostics and therapeutics has proceeded to date in a near vacuum of information about their structures. Here we report the first atomic structures of small molecules bound to amyloid. These are of the dye orange-G, the natural compound curcumin, and the Alzheimer's diagnostic compound DDNP bound to amyloid-like segments of tau and amyloid-β. The structures reveal the molecular framework of small-molecule binding, within cylindrical cavities running along the β-spines of the fibers. Negatively charged orange-G wedges into a specific binding site between two sheets of the fiber, combining apolar binding with electrostatic interactions, whereas uncharged compounds slide along the cavity. We observed that different amyloid polymorphs bind different small molecules, revealing that a cocktail of compounds may be required for future amyloid therapies. The structures described here start to define the amyloid pharmacophore, opening the way to structure-based design of improved diagnostics and therapeutics.

## Introduction

The challenge of developing chemical interventions for Alzheimer's disease has proceeded in a virtual vacuum of information about the three-dimensional structures of the two proteins most widely accepted as being involved in the etiology. These are amyloid-beta (Aβ) and tau [1],[2]. Both convert from largely natively disordered, soluble forms to toxic oligomers and fibers [2],[3] that may be related in structure [4]. Indeed, analogs of the well-established ligands to amyloid fibers, congo-red and thioflavin T, also bind Aβ oligomers labeling them in vitro and in vivo [5]. Screens of chemical libraries have uncovered dozens of small molecules that interact with amyloid [6]–[8]. Curcumin and various antibiotics are a few of many fiber inhibitors that also inhibit oligomer formation [7],[9],[10], supporting a common underlying structure in fibers and oligomers. Despite this progress, until now there have been no atomic-level structures showing how small molecules bind to amyloid and, consequently, no means for structure-based design of specific binders.

More is known about the molecular structure of amyloid fibers, both those associated with Alzheimer's disease and with the numerous other amyloid conditions [11]–[15]. Common to all amyloid fibers is their X-ray fiber-diffraction pattern, with two orthogonal reflections at about 4.8 Å and 10 Å spacing suggesting a “cross-β structure” [16],[17]. The determination of the first amyloid-like atomic structures revealed a motif consisting of a pair of tightly mated β-sheets, called a “steric zipper,” which is formed from a short self-complementary segment of the amyloid-forming protein [12],[18],[19]. The steric zipper structures elucidate the atomic features that give rise to the common cross-β diffraction pattern, corresponding to the 4.8 Å spacing between strands forming β-sheets and the ∼10 Å spacing between two mating β-sheets. The structures imply that stacks of identical short segments form the “cross-β spine” of the protofilament, the basic unit of the mature fiber, while the rest of the protein adopts either native-like or unfolded conformations peripheral to the spine [12],[20].

The short segments forming steric zippers, when isolated from the rest of the protein, form well-ordered fibers on their own, with essentially all properties of the fibers of their full-length parent proteins [21],[22]. These properties include similar fiber diameters and helical pitch, similar cross-β diffraction patterns, similar fiber-seeding capacities, similar stability, and similar dye binding. That stacked short amyloidogenic segments can constitute the entire spine of an amyloid-like fiber has been demonstrated with the enzyme RNase A, containing an insert of a short amyloidogenic segment [20],[23]. These RNase A fibers retain enzymatic activity, showing that native-like structure remains intact with only the stacked segments forming the spine. Thus while short amyloidogenic segments cannot recapitulate the entire complexity of their parent proteins, they nonetheless serve as good models for full amyloid fibers [24] and offer the informational advantage that they often grow into microcrystals whose atomic structures can be determined [12]. To date, structures for over 50 such steric zippers have been determined from a variety of disease-associated proteins ([18],[19],[25]–[27] and Colletier et al. unpublished results).

Here we use one such amyloid-forming segment from Aβ and one from tau to form co-crystals with low molecular weight compounds, with the aim of illuminating the nature of interactions of small molecules with amyloid. These complexes reveal a molecular framework which partially defines the amyloid pharmacophore, the structural features responsible for the binding of small molecules to amyloid aggregates.

## Results

### Screening for Co-Crystals of Amyloid-Like Segments with Small Molecules

In our attempts to obtain complexes of small molecules with amyloid-like segments from disease-related proteins, we screened for co-crystals grown from dozens of mixtures (Table S1). The majority of the resulting crystals yielded X-ray diffraction too poor for structure determination. Others led to structure determinations of the small molecule or amyloid-like segment alone. Out of hundreds of co-crystallization trials (Table S1), four mixtures, described below, yielded co-crystals with suitable X-ray diffraction from segments of Aβ and tau with amyloid binders.

### Crystal Structure of the KLVFFA Segment from Aβ Complexed with Orange-G

**Figure 1:**
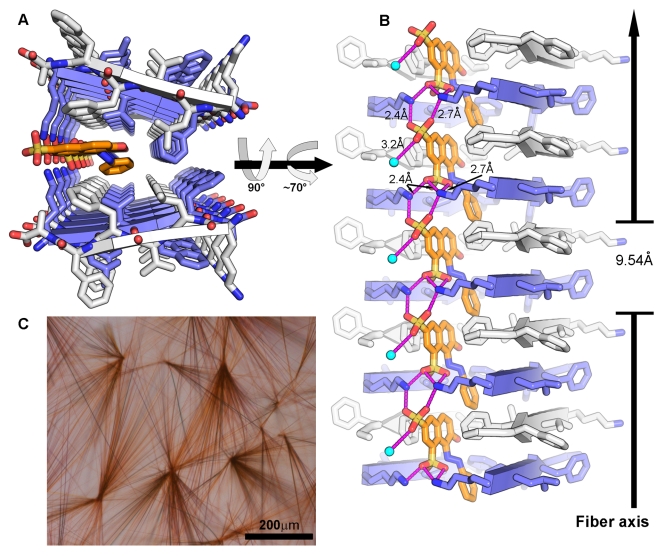
The crystal structure of the KLVFFA segment from Aβ complexed with orange-G. (A–B) The KLVFFA segments are packed as pairs of β-sheets forming the basic unit of the fiber, namely the steric zipper [12],[18]. Here 10 layers of β-strands are depicted; actual fibers contain ∼100,000 layers. Orange-G (orange carbons) wedges open the zipper and binds between the pair of β-sheets. KLVFFA and orange-G are shown as sticks with non-carbon atoms colored by atom type. The β-sheets are composed of anti-parallel strands (cartoon arrows), alternately colored white and blue. In panel A, the view looks down the fiber axis. In panel B, the view is perpendicular to the fiber axis; the β-strands run horizontally. The sulfonic acid groups of orange-G form salt links (pink lines) with four lysine residues, two protruding from each facing β-sheet and with a water molecule shown as an aqua sphere. Only side chain atoms are shown. The unit cell dimension of the crystal along the fiber axis (9.54 Å) is indicated. (C) Micro-crystals of KLVFFA co-crystallized with orange-G. More extensive packing interactions in larger regions of the crystal are presented in Figure S2.

**Figure 2:**
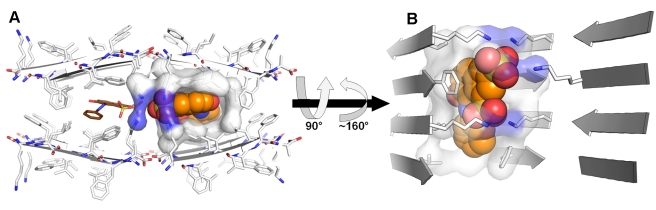
Binding cavities of orange-G within fibers of the KLVFFA segment from Aβ. Orange-G is bound internally to the steric zipper of KLVFFA (residues 16–21 of Aβ). It also contacts the lysine residues in the adjacent zipper. Peptide segments, forming β-sheet structures, are shown as arrows and sticks, colored by atom type with carbons in white. Orange-G carbons are in orange for one molecule and brown for the other molecule. Surface is shown for peptide atoms contacting the orange-G molecule with the orange carbons, shown as spheres. The view in (A) looks down the fiber axis. The view in (B) is perpendicular to the fiber axis. Only side chains of interacting residues are shown. The area of the fiber buried by orange-G (Methods) is 271 Å^2^ and 272 Å^2^ for the orange and brown colored orange-G, respectively, and is about 80% hydrophobic (contributed by the side chains of Leu17, Val18, Phe19, and Phe20). The polar interactions are contributed by the charged side chains of Lys16. In (B), one of the β-sheets from the adjacent pair and one orange-G molecule are removed for clarity.

**Figure 3:**
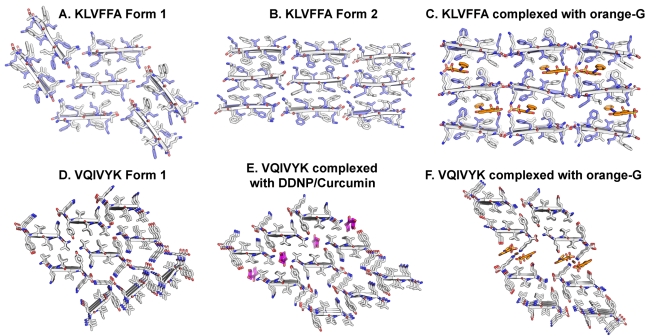
Small molecule binding is specific for fiber polymorphism. Three forms of the KLVFFA segment from Aβ (A–C) and the VQIVYK segment from the tau protein (D–F) are presented. These forms serve as examples of packing polymorphism observed for amyloid fibrils [25]. The view looks down the fiber axis; three layers of depth are depicted. The peptide segments and the small molecules are shown as sticks with non-carbon atoms colored by atom type. In the KLVFFA forms (A–C), the anti-parallel strands (cartoon arrows) are alternately colored white and blue. The VQIVYK forms (D–F) pack in parallel β-sheets (represented as cartoon arrows with white carbons). KLVFFA Form-1 (A) and Form-2 (B) (Colletier et al. unpublished results) and VQIVYK Form-1 (D) [18] are tightly packed such that there are no voids to accommodate binding of small molecules. VQIVYK Form-2 (E) [25] shows a shift in the steric zipper generating a void that can accommodate the binding of apolar molecules such as curcumin and DDNP. A docked model of curcumin binding is shown (colored magenta) (Methods). Orange-G binds to unique forms of both KLVFFA and VQIVYK (C and F), in which large voids are present in the crystal packing. In the KLVFFA complex (C), the binding is internal to the steric zipper, whereas in the VQIVYK complex (F), the binding is between pairs of β-sheets, i.e., internal to bundles of protofilaments.

The KLVFFA segment (residues 16–21) from Aβ contains apolar residues that participate in a hydrophobic spine in Aβ fibers and itself acts as an inhibitor of Aβ fibrillation [28],[29]. We previously determined the atomic structure of the KLVFFA segment in three crystal forms; all show the common steric zipper motif associated with amyloid fibers (Colletier et al. unpublished results). Orange-G (Figure S1), a synthetic azo dye used in histological staining, affects the formation of Aβ fibers [7]. The co-crystallization of KLVFFA with orange-G resulted in deeply colored crystals (Figure 1C). Mass spectrometric analyses of the crystals showed high abundance of orange-G (∼1∶1 molar stoichiometry with KLVFFA). Determination of the structure revealed a novel, fourth form of the KLVFFA steric zipper, with orange-G wedged between the paired β-sheets of the zipper, leading to partial opening of the zipper (Figures 1 and S2). Stabilization of the binding arises from packing of the aromatic rings of orange-G against the apolar, partially aromatic spine of KLVFFA (Figure 2). At the interface between orange-G and KLVFFA, a total of 500 Å^2^ of apolar surface area is covered, corresponding very roughly to a binding energy of 9 kcal/mol, or a dissociation constant of ∼0.3 µM (Methods). Further stabilization arises from the salt links between the negatively charged sulfonic acid groups of orange-G and positively charged lysine side chains from both β-sheets (Figures 1 and S2). Crystallization of KLVFFA under identical conditions but without orange-G resulted in the formation of colorless crystals with a structure similar to Form-1 (Colletier et al. unpublished results and Figures 3A and S3). Thus the binding of orange-G wedges apart the previously tightly mating pair of sheets of the steric zipper.

All four crystal forms of KLVFFA, including the complex with orange G, show an anti-parallel β-strand stacking in the steric zipper (Colletier et al. unpublished results and Figure 1). Nuclear magnetic resonance (NMR) characterization of Aβ fibers suggested a parallel orientation of the full-length Aβ [30]. Yet an anti-parallel orientation was proposed for various Aβ segments, both in the region of the KLVFFA segment (residue numbers are indicated in subscript): Aβ_16–22_ [31], Aβ_17–21_ [32], and Aβ_11–25_ [32], as well as for a segment at the C-terminus: Aβ_34–42_ [33]. Moreover, the “Iowa” Aβ mutant that is related to a familial, early onset, Alzheimer's disease [34] also displays an anti-parallel β-strand orientation. Of potential importance, Aβ oligomers were also suggested to form anti-parallel β-sheet structures [35],[36].

### Crystal Structures of the VQIVYK Segment from the Tau Protein with Orange-G

**Figure 4:**
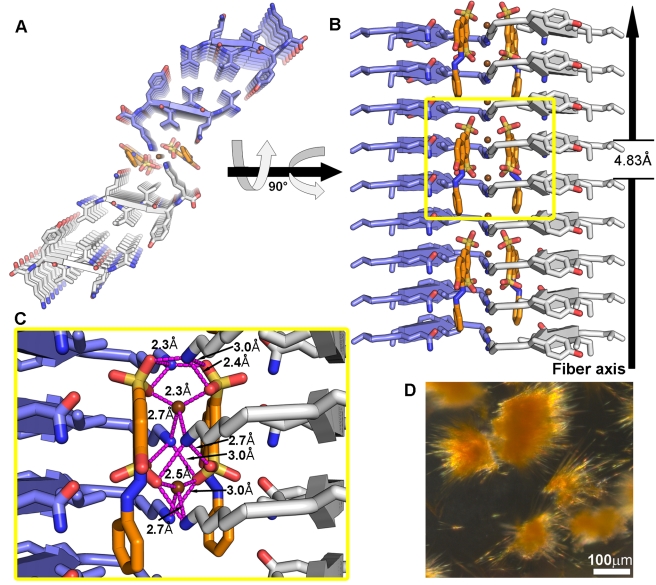
The crystal structure of the VQIVYK segment from the tau protein complexed with orange-G. (A–C) The VQIVYK segments pack in parallel, in-register β-sheets (cartoon arrows) that form steric zippers (two zippers are shown in panel A). Nine layers of the fiber are depicted. VQIVYK and orange-G are shown as sticks with non-carbon atoms colored by atom type. The carbons of VQIVYK are colored white for one steric zipper and blue for the other. Two orange-G molecules (orange carbons) mediate contacts between two pairs of steric zippers; that is, orange-G is located between the protofilaments composing the fiber. In panel A, the view looks down the fiber axis. In panel B, the view is perpendicular to the fiber axis. Only the two sheets that are in contact with orange-G are shown. Backbone atoms are not shown. The unit cell dimension of the crystal along the fiber axis (4.83 Å) is indicated. The length of orange-G spans multiple unit cells of the fibril; that is, the dimensions of the small molecule and the fibril unit cell are incommensurate (see Text S1). Panel C is an inset of panel B, focusing on the network of salt links (pink lines) between the sulfonic acid groups of two orange-G molecules and six lysine residues and with zinc cations (brown spheres). (D) Micro-crystals of VQIVYK co-crystallized with orange-G.

**Figure 5:**
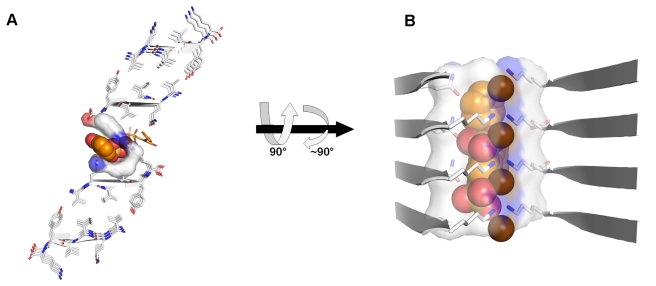
Binding cavities of orange-G within fibers of the VQIVYK segment from the tau protein. Orange-G is bound between steric zippers of VQIVYK, i.e., internally to a bundle of protofilaments. The VQIVYK segment is located at the third repeat of the tau protein. Since there are many isoforms of tau, we will number the VQIVYK residues 1–6 for simplicity. Peptide segments, forming β-sheet structures, are shown as arrows and sticks, colored by atom type with carbons in white. Orange-G carbons are in orange. Surface is shown for peptide atoms contacting the orange-G molecule (shown as spheres). The view in (A) looks down the fiber axis. The view in (B) is perpendicular to the fiber axis. Only side chains of interacting residues are shown. The area of the fiber buried by orange-G (Methods) is 309 Å^2^ and about 40% hydrophobic (contributed by the side chains of Val4 and the carbon chain of Lys6) and 60% polar (contributed by Gln2, Lys6, and the C-terminus). In (B), only one orange-G molecule and the β-sheets directly contacting it are shown. Zinc atoms are shown as brown spheres. It is noteworthy that interactions between lysine residues, zinc cations, and negatively charged groups are a motif observed in the Protein Data Bank; for example see [72].

The VQIVYK segment of tau was suggested as the minimal interaction motif for fiber formation [37]. We previously determined the crystal structure of VQIVYK in two crystal forms; both show the common steric zipper motif of amyloid fiber-like structures [18],[25]. Co-crystallization of VQIVYK with orange-G resulted in deep orange crystals (Figure 4D). Mass spectrometric analyses of the crystals showed relatively high abundance of orange-G (∼1∶10 molar stoichiometry with VQIVYK). Determination of the structure revealed a new crystal form of VQIVYK (Figure 4). Similar to Form-1 [18], the steric zipper shows a tight and dry interface; yet there is a large void between pairs of steric zipper, in contrast to the tightly packed structure of Form-1 (Figure 3D). Orange-G is situated within this void, binding between lysine side chains facing each other from two parallel pairs of zippers, forming an electrostatic network that also involves zinc cations (Figures 4–5). As in its complex with KLVFFA, orange-G lies with its long axis parallel to the fiber axis.

Crystallization of VQIVYK alone, under identical conditions to the co-crystallization of the VQIVYK-orange-G mixture, resulted in the formation of colorless fibrous crystals (Figure S3) giving poor X-ray diffraction. Under these conditions, the presence of orange-G appears crucial for the formation of well-ordered crystals.

### Crystal Structures of the VQIVYK Segment from the Tau Protein with Curcumin and DDNP

**Figure 6:**
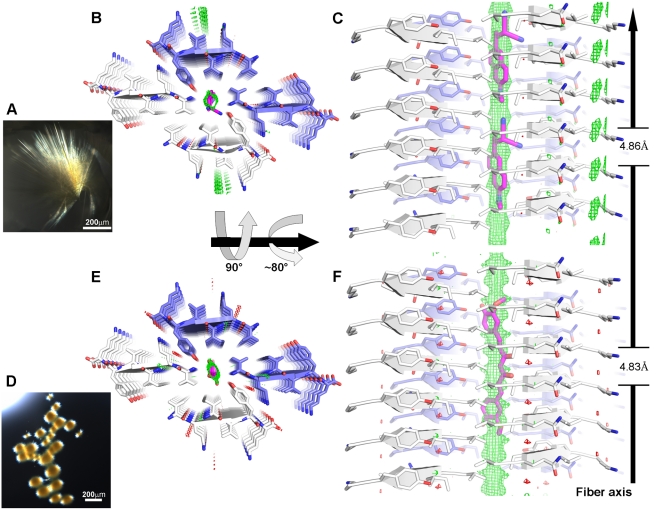
Models of DDNP and curcumin bound to the VQIVYK fiber based on undifferentiated electron density. Panels A and D are micro-crystals of VQIVYK co-crystallized with DDNP and curcumin, respectively. In the structure of the complexes with DDNP (B–C) and curcumin (E–F), VQIVYK is packed in a form having a steric zipper with one β-sheet shifted in relation to the other β-sheet (cartoon arrows). The carbons of VQIVYK are colored white for one steric zipper and blue for the other. VQIVYK, DDNP, and curcumin are shown as sticks with non-carbon atoms colored by atom type. Six layers of the fiber are depicted. In panels B and E, the view looks down the fiber axis. In panels C and F, the view is perpendicular to the fiber axis. In both complexes, only the VQIVYK segment is modeled into the electron density, and in both, there is an apparent difference electron density Fo-Fc map (shown as mesh, +3σ in green and −3σ in red) located in the void formed by the shift of the steric zipper. The positive density (part of the structure that has not been modeled, green mesh) displays a continuous tube-like shape, running along the fiber axis. We attribute this density to the presence of the small molecules, yet it is too undifferentiated to fit atoms in detail. DDNP (B–C, two molecules are shown) and curcumin (E–F) (both in magenta carbons) have been computationally docked (Methods) into the structures and fit the location of the positive density. The unit cell dimension of the crystal along the fiber axis is indicated. The length of both DDNP and curcumin spans multiple unit cells of the fibril; that is, the dimensions of the small molecule and the fibril unit cell are incommensurate (see Text S1).

**Figure 7:**
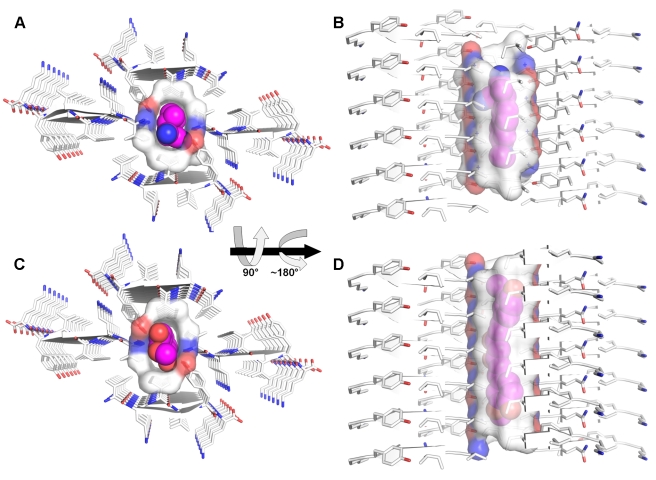
Binding cavities of DDNP or curcumin within fibers of the VQIVYK segment from the tau protein. VQIVYK segments, forming parallel β-sheet structures, are shows as arrows and sticks, colored by atom type with carbons in white. Docked DDNP (A and B) and curcumin (C and D) are shown as spheres with carbons colored magenta. Both small molecules are bound in the void formed within two shifted steric zippers. Surface is shown for peptide atoms contacting the small molecules. The area of the fiber buried by DDNP or curcumin is 242 Å^2^ or 351 Å^2^, respectively, and is about 50% hydrophobic (contributed by the side chain of Val1 and Ile3) and 50% polar (contributed by the hydroxyl of Tyr5 and the N-termini). The view in panels A and C looks down the β-sheets (fiber axis). The view in panels B and D is perpendicular to the fiber axis, with β-strands running horizontally.

Curcumin (Figure S1) from the plant turmeric protects neuronal cells against amyloid toxicity [9]. DDNP (Figure S1) [38] and its analogs, synthetic diagnostics, bind Alzheimer's-associated neurofibrillary tangles and β-amyloid senile plaques and are used for the detection of plaques in the brains of Alzheimer's disease patients [39],[40]. Co-crystallization of VQIVYK with either curcumin or DDNP resulted in yellowish crystals (Figure 6). Similar to Form-2 of VQIVYK [25], the structures of VQIVYK complexed with either curcumin or DDNP revealed that in both complexes, each member of a pair of β-sheets is shifted relative to the other, partially eliminating the dry interface in the steric zipper structure (Figures 3E and 6). In both complexes, the electron density attributed to the small molecule (either curcumin or DDNP) lies along the void left by the shifting of the steric zipper. This electron density is too undifferentiated to model the small molecule in atomic detail. However, it shows that the long axes of both curcumin and DDNP lie parallel to the fiber axis (Figures 6–7), as in the KLVFFA and VQIVYK structures with orange-G.

Despite the lack of differentiated electron density for curcumin and DDNP in VQIVYK, there is strong evidence for the presence of the small molecules in the crystals. The crystals show a distinctive color, whereas the control crystals (grown under identical condition without the small molecule) are colorless (Figure S4). The control crystals also lack the additional positive density attributed to the small molecule (Figure S4). Co-crystals of VQIVYK and DDNP grown under alternative crystallization conditions showed a similar positive electron density (Figure S4D), supporting its attribution to DDNP. Furthermore, the crystals grown in the presence of DDNP appeared within days, whereas the control crystals grew only after 8 months, suggesting that the presence of DDNP is a catalyst for crystallization. The strongest evidence supporting the presence of the small molecules in the structure is provided by mass spectrometric analyses of the crystals. The analyses also provided the reasoning for the undifferentiated electron density, showing a very low molar abundance of both curcumin and DDNP in the crystal (∼100 and ∼400 VQIVYK segments for each curcumin or DDNP molecule, respectively), which is in close approximation to the experimentally established molar ratio between FDDNP (the fluoridated version of DDNP) (Figure S1) and Aβ fibril of 1∶1500 to 1∶3000 [41]. We conjecture that the lack of site anchoring of the hydrophobic, uncharged small molecules to specific residues in the fibril leads to undifferentiated electron density. Furthermore, the nature of the binding site (a narrow tube running along the β-sheets) (Figures 6–7) implies that the apolar small molecules are free to drift along the fiber axis (Text S1).

The common feature of the structures of four amyloid/small-molecule complexes is that the small molecules bind to fibers in a similar orientation, along the β-sheets, with their long axes parallel to the fiber axis. This orientation was previously proposed for the binding of thioflavin T to bovine insulin and bovine β-lactoglobulin amyloid fibrils using polarized laser confocal microscopy [42]. A similar mode of binding was seen in co-crystals of oligomer-like β-2-microglubulin with thioflavin T, showing that thioflavin T is bound between β-sheets, orthogonal to the β-strands [43]. The orientation of congo-red was also suggested to be parallel to the amyloid long axis based on electron diffraction, linear dichroism [44], and a recent NMR-based model of congo-red bound to the fungal prion domain HET-s(218–289) [45].

## Discussion

Our crystal structures of small molecules bound within amyloid-like steric zippers begin to define the molecular frameworks, or pharmacophores, for the design of diagnostics and drugs for Alzheimer's and other aggregation diseases. The amyloid components in our structures are steric zippers formed by stacks of six-residue segments from Alzheimer-related proteins. Although these steric zippers cannot represent all aspects of the full-length amyloid parent proteins, they share many properties and are commonly used as models of the amyloid β-spine and of aggregation [22],[24]. The small molecules in our structures bind along the β-spine, and because the parent amyloids contain the same segments, we expect a similar mode of binding along the spine of the full-length parent amyloid fibers. Moreover, we anticipate the steric zipper spine of the parent fibers to be flanked with the rest of the protein residues in a native-like or unfolded conformation [12],[20] and therefore to contain more solvent channels, or accessible sites for the binding of the small molecules, compared to the very compact packing of the steric zipper segments. Consistently, orange-G, curcumin, and DDNP all bind to, or affect fibrillation of, full-length fibers [7],[9],[39].

### Molecular Frameworks of Amyloid Binders

Overall, the complexes presented here suggest the nature of two molecular frameworks for the binding of small molecules to amyloid fibers. The first molecular framework pertains to site-specific binders, such as charged compounds that form networks of interactions with sequence motifs, and is relatively well defined. The second molecular framework, far less well defined at this point, pertains to broad-spectrum binders, such as uncharged aromatic compounds that bind to tube-like cavities between β-sheets. For binding amyloid deposits in the brain, uncharged molecules could be more effective because of superior blood-brain-barrier penetrability. The same frameworks, offering cavities along β-sheets, might also exist in amyloid oligomers known to be rich in β-sheets and possibly fiber-like [46], similar to the observed binding of amyloid markers to β-sheets in non-fibrillar structures [43],[47]. Consistent with this, both oligomers and fibers are inhibited by similar compounds, including curcumin [7],[9].

The specific binding of orange-G allows definition of the chemical properties of a specific molecular framework. The prominent feature of amyloid structures is the separation of β-strands (forming a β-sheet) by ∼4.8 Å. In structures with strands packed in an antiparallel orientation, as observed for the KLVFFA fibers and for a rare mutation in Aβ that is associated with massive depositions of the mutant protein and early onset of the disease [34],, the separation of repeating units (2 strands) is twice as great, ∼9.6 Å. Orange-G contains two negatively charged sulfonic acid groups facing the same direction, with the sulfur atoms spaced ∼5 Å apart and the oxygen atoms separated by 4.5–7.5 Å. This framework allows the formation of salt links between the sulfonic acid groups and lysine ammonium ions from every repeating strand in both KLVFFA (anti-parallel orientation) and VQIVYK (parallel orientation) fibers (Figures 1 and 4). This shows that a specific framework includes two charged moieties spaced either ∼4.8 Å or ∼9.6 Å apart. The specific sequence motif of the spine of the fiber and the separation of the β-strands dictates the signs of the necessary charges in the small molecule and their separation.

Within our framework, an apolar aromatic spine is another essential moiety [22]. The largely apolar KLVFFA segment attracts the apolar surface of orange-G, stabilizing the binding (Figure 2). In the complex with VQIVYK, the aromatic rings of orange-G are also packed against apolar side chains, but the binding is largely mediated via polar interactions with glutamine and lysine side-chains at the edges of two steric zippers (Figure 5). The differences in the binding cavities between the KLVFFA and VQIVYK fibers may account for the higher molecular stoichiometry within the KLVFFA-orange-G crystals observed by mass spectrometric analyses, and the correspondingly greater order of this complex (Figures S5–S6).

Despite the lack of atomized electron density for the binding of curcumin and DDNP in VQIVYK fibers, the location of the binding cavity is clear. It is narrow, restricting rotation of the small molecule (Figures 6–7). The atomic groups lining the binding cavity are about half apolar and half polar (Figure 7). The tube-like shape favors the binding of uncharged molecules, such as DDNP and curcumin. The binding site is, however, insufficiently site-specific to allow for high occupancy and ordered interactions and is not yet well defined in atomic detail.

Our structures show that different small molecules bind along the β-spine of amyloid-like fibers. In case fibers contain more than a single spine, the molecules might bind to multiple sites. This is more likely for the broad-spectrum hydrophobic compounds but can also apply for charged compounds. For example, we observed orange-G to bind to two different steric zippers, of KLVFFA and VQIVYK, with the commonality of binding to lysine side chains protruding from the β-sheets.

Congo-red, a known amyloid marker, contains two sulfonic acid groups, similar to orange-G, but they are spaced ∼19 Å apart, which might account for its lack of specificity [44]. In a recent model, built using NMR constrains, congo-red was computationally docked to the fungal prion domain HET-s(218–289), suggesting that the sulfonic acid groups interact with lysine residues protruding from the sheets [45], similar to orange-G in our structures. However, in the model, the strands of HET-s are arranged in an anti-parallel orientation and the sulfonic acid groups of congo-red interact with every other lysine along the fiber [45], while orange-G interacts with every single lysine in both the KLVFFA and VQIVYK complexes (Figures 1 and 4). Both congo-red and thioflavin T, another known marker, bind to numerous different β-structures, even in a non-fibrillar form [43],[47]. Despite their limited specificity and low affinity [49],[50], these dyes play a major role in amyloid research because their binding is detectible via birefringence or fluorescence [51],[52]. An important application of our structures is for the design of new markers for aggregation that will be more potent and can also be used in vivo.

### The Two Molecular Frameworks and Function

Defining these two molecular frameworks illuminates functional attributes of specific and broad-spectrum amyloid binders. This distinction is consistent with competitive kinetic experiments demonstrating that the binding of FDDNP (the fluoridated analog of DDNP) to Aβ fibrils is displaceable by the uncharged non-steroidal anti-inflammatory naproxen, but not by the common charged dyes congo-red and thioflavin T [53]. Moreover, in vitro FDDNP labels amyloid-like structures in a fashion similar to congo-red and thioflavin T, providing further evidence for the broad-spectrum type of binding [54]. Knowledge of both frameworks can lead to the design of more potent and specific compounds. These molecules can act as binders and be used as diagnostics, or serve as inhibitors of aggregation by either destabilizing steric zippers by wedge action (Figure 1) or binding between steric zippers preventing higher-order β-sheet interactions (Figure 4).

In the case of the complexed curcumin and DDNP structures, we hypothesize that the tube-like cavity along the β-sheets provides an adequate site for the binding of many compounds of similar properties. However, the lack of specific interactions allows the small molecule to drift along the fiber axis, leading to lower occupancy and a degree of fluidity in the structure. Extrapolating from our structures, we expect that various aromatic compounds, such as polyphenols [6], would bind to a variety of amyloid-forming sequences because of a cylindrical, partially apolar cavity that forms between the pairs of β-sheets forming the fibers. These cavities might also provide binding sites for various kinds of apolar drugs, such as benzodiazepines and anesthetics, explaining some of the altered pharmacokinetic properties and increased sensitivity detected in elderly [55].

A subtle implication of our structures for the design of effective therapeutic treatments is the specificity they reveal of ligand binding to particular fiber polymorphs (Figure 3). Various amyloid proteins show diverse fiber morphologies that are correlated with different patterns of pathology and toxicity [56],[57]. In earlier work, we have suggested that fiber polymorphism has its molecular basis in different steric zippers (β-sheet packing) formed by the same sequence [25]. Our new findings show that different compounds bind to different fiber polymorphs formed by the same sequence. For example, orange-G displaces one VQIVYK zipper relative to its mate; that is, wedges between protofilaments (Figures 3F and 4). In contrast, both DDNP and curcumin opportunistically bind to cylindrical cavities at the edges of VQIVYK zippers, in a void formed within a different VQIVYK β-sheet packing (Figures 3E and 6). This suggests that each compound binds to only a sub-population of fibers. Thus, just as cocktails of anti-HIV drugs are necessary to inhibit different viral strains, a combination of compounds may be necessary to bind to the several amyloid polymorphs present.

### Conclusions

Four crystal structures of small molecules bound to fiber-forming segments of the two main Alzheimer's disease proteins show common features. The small molecules bind with their long axes parallel to the fiber axis. The structures reveal a sequence-specific binder which forms salt links with side-chains of the steric zipper spines of the fibers and non-specific binders which lie in cylindrical cavities formed at the edges of several steric zippers. Small-molecule binding is specific to particular steric-zipper polymorphs, suggesting that effective Alzheimer's diagnostics and therapeutics may have to be mixtures of various compounds to bind to all polymorphs present. The complexes presented here suggest routes for structure-based design of combinations of compounds that can bind to a spectrum of polymorphic aggregates, to be used as markers of fibers and as inhibitors of aggregation.

## Materials and Methods

### Peptide and Compounds

Peptide segments (custom synthesis) were purchased from CS Bio. Orange-G and curcumin were purchased from Sigma-Aldrich. DDNP was synthesized as described in [38],[58].

### Crystallizing Conditions

All crystals were grown at 18°C via hanging-drop vapor diffusion. All crystals appeared within 1 wk, except the negative control crystals of VQIVYK+DDNP that took 8 mo to grow.

#### VQIVYK+orange-G

The drop was a mixture of 10 mM VQIVYK and 1 mM orange-G in water, and reservoir solution (0.1 M zinc acetate dehydrate, 18% polyethylene glycol 3350). The structure was solved to 1.8 Å resolution and contained one segment, one orange-G, two water molecules, two zinc atoms, and one acetate molecule in the asymmetric unit.

#### VQIVYK+DDNP

The drop was a mixture of 6 mM VQIVYK and 1 mM DDNP in 60% ethanol, and reservoir solution (0.52 M potassium sodium tartrate, 0.065 M HEPES-Na pH 7.5, 35% glycerol). The structure was solved to 1.2 Å resolution and contained one segment and three water molecules in the asymmetric unit.

#### VQIVYK+DDNP from second crystallization conditions

The drop was a mixture of 6 mM VQIVYK and 1 mM DDNP in 60% ethanol, and reservoir solution (1.2 M DL-malic acid pH 7.0, 0.1 M BIS-TRIS propane pH 7.0). The structure was solved to 1.65 Å resolution and contained one segment, and three water molecules in the asymmetric unit.

#### Negative control crystals to VQIVYK+DDNP

The drop was a mixture of 6 mM VQIVYK in 60% ethanol and reservoir solution (0.52 M potassium sodium tartrate, 0.065 M HEPES-Na pH 7.5, 35% glycerol). The structure was solved to 1.2 Å resolution and contained one segment, and one water molecule in the asymmetric unit.

#### VQIVYK+curcumin

The drop was a mixture of 10 mM VQIVYK and 1 mM curcumin in 80% dimethyl sulfoxide (DMSO), and reservoir solution (0.1 M Tris.HCl pH 8.5, 70% (v/v) MPD (2-methyl-2,4-pentanediol)). The structure was solved to 1.3 Å resolution and contained one segment, and two water molecules in the asymmetric unit.

#### Negative control crystals to VQIVYK+curcumin

The drop was a mixture of 10 mM VQIVYK in 80% DMSO and reservoir solution (0.1 M Tris.HCl pH 8.5, 70% (v/v) MPD (2-methyl-2,4-pentanediol)). The structure was solved to 1.3 Å resolution and contained one segment, and one water molecule in the asymmetric unit.

#### KLVFFA+orange-G

The drop was a mixture of 10 mM KLVFFA and 1 mM orange-G in water, and reservoir solution (10% w/v polyethylene glycol 1,500, 30% v/v glycerol). Another drop was a mixture of 5 mM KLVFFA and 1 mM orange-G in water, and reservoir solution (30% w/v polyethylene glycol 1,500, 20% v/v glycerol). The structure was solved to 1.8 Å resolution and contained four segments, two orange-G molecules, and 11 water molecules in the asymmetric unit.

#### Negative control crystals to KLVFFA+orange-G

The drop was a mixture of 10 mM KLVFFA in water, and reservoir solution (10% w/v polyethylene glycol 1,500, 30% v/v glycerol). Another drop was a mixture of 5 mM KLVFFA in water, and reservoir solution (30% w/v polyethylene glycol 1,500, 20% v/v glycerol). The structure was solved to 2.1 Å resolution and contained one segment and three water molecules in the asymmetric unit.

### Structure Determination and Refinement

X-ray diffraction data were collected at beamline 24-ID-E of the Advanced Photon Source (APS), Argonne National Laboratory; wavelength of data collection was 0.9792 Å. Data were collected at 100 K. Molecular replacement solutions for all segments were obtained using the program Phaser [59]. The search models consisted of available structures of the same segment or geometrically idealized β-strands. Crystallographic refinements were performed with the program Refmac5 [60]. Model building was performed with Coot [61] and illustrated with PyMOL [62]. There were no residues that fell in the disallowed region of the Ramachandran plot. Simulated annealing composite omit map was generated using CNS [63],[64]; 10% was omitted.

### Computational Docking

Three-dimensional (3-D) structures of the small molecules were generated using Corina (Molecular Networks; http://www.molecular-networks.com/online_demos/corina_demo) and Chemical Identifier Resolver (http://cactus.nci.nih.gov/translate/). Additional 3-D conformations were generated using OpenEye Omega [65]. The small molecule was placed in approximate location according to the electron density map. The small molecule was docked to the peptide fibrillar structure using RosettaLigand [66],[67]. The protein side chains were fixed. The generated docked structures (1,000 for KLVFFA-orange-G and 500 for the rest of the structures) were further refined using Refmac5 [60] and the 10 best structures (based on lowest free-R [68]) were analyzed and showed to be very similar to each other. The best structures were further optimized and refined and the one with the lowest free-R was chosen as the final structure.

### Solvent Accessible Surface Area, Free Energy, and Dissociation Constant Calculations

The area buried of the small molecules within the fiber structure was calculated using Areaimol [69],[70] with a probe radius of 1.4 Å. The difference between the accessible surface areas of the fiber structure alone and with the small molecule constitutes the reported area buried. The Areaimol [69],[70] calculations were also used to report the segment atoms that are in contact with the small molecules (shown in Figures 2, 5, and 7), and the percentage of apolar and polar contacts.

Binding energy and corresponding dissociation constant of one orange-G molecule to the KLVFFA fiber were estimated from the apolar surface area (contributed by carbon atoms) that is covered by the interaction and was calculated using Areaimol [69],[70]. The difference between the apolar accessible surface areas of the fiber structure alone and with the small molecule was added to the difference between the apolar accessible surface areas of the small molecule alone and with the fiber. These calculations resulted in 500 Å^2^ of apolar surface area covered. The binding energy was calculated from the formula [71] *ΔG^0^ = 18 cal*×*Å* ^−*2*^×*mol* ^−*1*^ = 18×500 cal/mol  = 9 kcal/mol. The dissociation constant was calculated from *ΔG^0^ = *−*RTlnK.* Thus, *K = * exp(−*ΔG/RT*)  = 3×10^−7^ M  = 0.3 µM.

### Mass Spectrometry Analysis of the Co-Crystals

Liquid chromatography tandem mass spectrometry (LC-MSMS) was used to measure the molar ratios of the peptide segments and the small molecules within the crystals. Authentic samples of the peptides and each of the small molecules were used to prepare standard response curves. Crystals from each of the four mixtures of peptides and small compounds were individually picked (using a sharpened glass capillary) and re-dissolved in 5%–10% acetonitrile. The samples were divided into two aliquots, one for the peptide analyses and the other for the small molecule analyses, and the amount of each component in the samples was interpolated using the standard curves.

Peptide standards (dry powder of VQIVYK and KLVFFA) were dissolved in water and prepared in concentrations ranging from 0.05 µM to 0.01 mM in 0.1% TFA. Aliquots of the standards and the re-dissolved crystals were separately injected (50 µL) onto a polymeric reverse phase column (PLRP/S, 2×150 mm, 5 µm, 300 Å; Varian) equilibrated in Buffer A (0.1% formic acid in water) and eluted (0.25 mL/min) with an increasing concentration of Buffer B (0.1% formic acid in acetonitrile). The effluent from the column was directed to an Ionspray source attached to a triple quadrupole mass spectrometer (Perkin Elmer/Sciex API III^+^) operating under previously optimized positive ion mode conditions. Data were collected in the positive ion multiple reaction monitoring (MRM) mode in which the intensity of specific parent→fragment ion transitions were recorded (VQIVYK, m/z 749.5→341.3, 749.5→409.4, 749.5→440.3, 749.5→522.5; KLVFFA, 724.4→84, 724.4→488.3, 362.7→84, 362.7→120.1).

Similar procedures were used for the analyses of the small molecules. Orange-G was dissolved in water and diluted with 10% ammonium acetate to concentrations ranging from 2 nM to 20 µM. Solutions of the standard and the re-dissolved crystals were separately injected (50 µL) onto a silica based reverse phase column (Supelco Ascentis Express C18, 150×2.1 mm, 2.7 µm) equilibrated in Buffer A (10 mM ammonium acetate) and eluted (0.2 mL/min) with an increasing concentration of Buffer B (acetonitrile/Isopropanol 1∶1 containing 10 mM ammonium acetate). The negative ion MRM transitions were m/z 407.1→302.1 and 407.1→222.1.

DDNP was dissolved in 95% ethanol and diluted with 10% ammonium acetate to concentrations ranging from 2 nM to 20 µM. Solutions of the standards and the re-dissolved crystals (further diluted with acetonitrile:methanol:water:acetic-acid (41∶23∶36∶1, v/v/v/v) to ensure dissolution) were separately injected (50 µL) onto a silica based reverse phase column (Supelco Ascentis Express C18, 150×2.1 mm, 2.7 µm) equilibrated in Buffer A (10 mM ammonium acetate) and eluted (0.2 mL/min) with an increasing concentration of Buffer B (acetonitrile/Isopropanol 1∶1 containing 10 mM ammonium acetate). The positive ion MRM transition was: DDNP − m/z 262.1→247.1.

Curcumin was dissolved and diluted in acetonitrile:methanol:water:acetic-acid (41∶23∶36∶1, v/v/v/v) to concentrations ranging from 2 nM to 2 µM. Aliquots of the standards and the re-dissolved crystals (further diluted with acetonitrile:methanol:water:acetic-acid (41∶23∶36∶1, v/v/v/v) to ensure dissolution) were injected (100 µL) onto a silica based reverse phase column (Waters Symmetry Shield RP18 5 µM, 3.9×150 mm) equilibrated in Buffer A (10 mM ammonium acetate) and eluted (0.5 mL/min) with an increasing concentration of Buffer B (acetonitrile/Isopropanol 1∶1 containing 10 mM ammonium acetate). The negative ion MRM transitions were m/z 367.1→173.1, 367.1→149.

### Accession Numbers

Structures of KLVFFA complexed with orange-G and VQIVYK complexed with orange-G are deposited in the Protein Data Bank (PDB) with accession codes 3OVJ and 3OVL, respectively (Table S2). The rest of the structures and crystallographic tables are accessible in http://people.mbi.ucla.edu/meytal/CoCrystalPaper/.

## Supporting Information

Figure S1 Chemical structures of the small molecule binders.
(TIF)

Figure S2 The crystal structure of the KLVFFA segment from Aβ complexed with orange-G shows extensive interactions between two orange-G molecules and the fiber. The KLVFFA segments are packed as pairs of β-sheets with orange-G bound internally to the steric zipper. The asymmetric unit of the crystal contains four peptide segments, two orange-G molecules, and 11 water molecules. Here, four layers of β-strands and two steric zippers are shown. KLVFFA and orange-G are shown as sticks with non-carbon atoms colored by atom type. The anti-parallel strands (cartoon arrows) are alternately colored white and blue, with the adjacent steric zipper colored in darker hues. The two orange-G molecules in the asymmetric unit, with carbons in orange and brown, display similar interactions with the fiber. In panel A, the view looks down the fiber axis. In panels B–C, the view is perpendicular to the fiber axis. The sulfonic acid groups of orange-G form salt links (pink lines) with five lysine residues, four protruding from facing β-sheets of the steric zipper and one from the adjacent zipper (only the latter is presented as pink lines in panel A). Only side chains of residues participating in salt links are shown. One of the sheets from the adjacent pair and one orange-G molecule are removed for clarity.
(TIF)

Figure S3 Crystals of the KLVFFA segment from Aβ and of the VQIVYK segment from the tau protein grown with and without orange-G. (A–B) Micro-crystals of the KLVFFA segment of Aβ grown under identical conditions (Methods) with (A) and without (B) orange-G. (C–D) Micro-crystals of the VQIVYK segment of the tau protein grown under identical conditions (Methods) with (C) and without (D) orange-G.
(TIF)

Figure S4 Crystal structures used as controls for the complexes of the VQIVYK segment from the tau protein with DDNP and curcumin. (A) Micro-crystals of VQIVYK co-crystallized with DDNP; the structure is shown in panel C. (B) Micro-crystals of VQIVYK crystallized under identical conditions to the crystals in panel A, lacking DDNP (Methods). The structure is shown in panel E. (D) The structure of VQIVYK co-crystallized with DDNP, grown under different crystallization conditions than the structure shown in panel C (Methods). (F) Micro-crystals of VQIVYK co-crystallized with curcumin; the structure is shown in panel G. (I) Micro-crystals of VQIVYK crystallized under identical conditions to the crystals in panel F, lacking curcumin (Methods). The structure is shown in panel H. In panels C–E and G–H, six layers of the VQIVYK fiber are depicted. The VQIVYK segment pack in parallel β-sheets (represented as cartoon arrows with white carbons). The view is perpendicular to the fiber axis, with β-strands running horizontally. Only the VQIVYK segment was modeled into the electron density. The difference electron density Fo-Fc map is shown as mesh (+3σ in green and −3σ in red), indicating missing atoms in the model. The crystals grown without the small molecule, either DDNP or curcumin (panels B and I, respectively), are colorless, whereas the co-crystals are colored (panels A and F, respectively). Moreover, the VQIVYK_apo_ structures also lack the positive density (part of the structure that has not been modeled, green mesh) that we attribute to the presence of the small molecule (panels E and H versus panels C and G, respectively). Both structures of VQIVYK complexed with DDNP, grown under different crystallization conditions (panels C and D), show a similar, tube-like, positive electron density map, supporting the attribution of DDNP to this density.
(TIF)

Figure S5 Electron density maps and simulated annealing composite omit maps of the KLVFFA segment from Aβ complexed with orange-G. The KLVFFA segments and orange-G molecules are shown as sticks with non-carbon atoms colored by atom type. The β-sheets are formed via stacks of anti-parallel strands, alternately colored with carbons in white and in blue. The carbons of the orange-G molecules are colored orange. Water molecules are shown as aqua spheres. The view here is perpendicular to the fiber axis. (A–C) The electron density 2Fo-Fc map is shown as grey mesh (1.3σ). The difference electron density Fo-Fc map is shown as mesh (+3σ in green and −3σ in red). (D–F) The simulated annealing composite omit 2Fo-Fc map (10% omitted) is shown as grey mesh (1.3σ). Panels B–C and D–E focus on the two orange-G molecules in the asymmetric unit.
(TIF)

Figure S6 Electron density map of the VQIVYK segment from the tau protein complexed with orange-G. The VQIVYK segment and orange-G are shown as sticks with carbon atoms colored grey and orange, respectively, and non-carbon atoms colored by atom type. The view in panel A looks down the fiber axis. The view in panel B is perpendicular to the fiber axis and focuses on orange-G. The electron density 2Fo-Fc map is shown as grey mesh (1.3σ). The difference electron density Fo-Fc map is shown as mesh (+3σ in green and −3σ in red).
(TIF)

Table S1 Screening for co-crystals from mixtures of amyloid-like segments with small molecules.
(PDF)

Table S2 Data collection and refinement statistics (molecular replacement).
(PDF)

Text S1 (I) Using computational docking for structure determination. (II) Incommensurate structures.
(PDF)

## Abbreviations

Aβ: amyloid-beta
NMR: nuclear magnetic resonance
