# Supplementary figures and images for: Towards a Pharmacophore for Amyloid
Source: PLoS Biol. 2011 Jun 14;9(6):e1001080. doi: 10.1371/journal.pbio.1001080 (PMC3114762; doi:10.1371/journal.pbio.1001080)

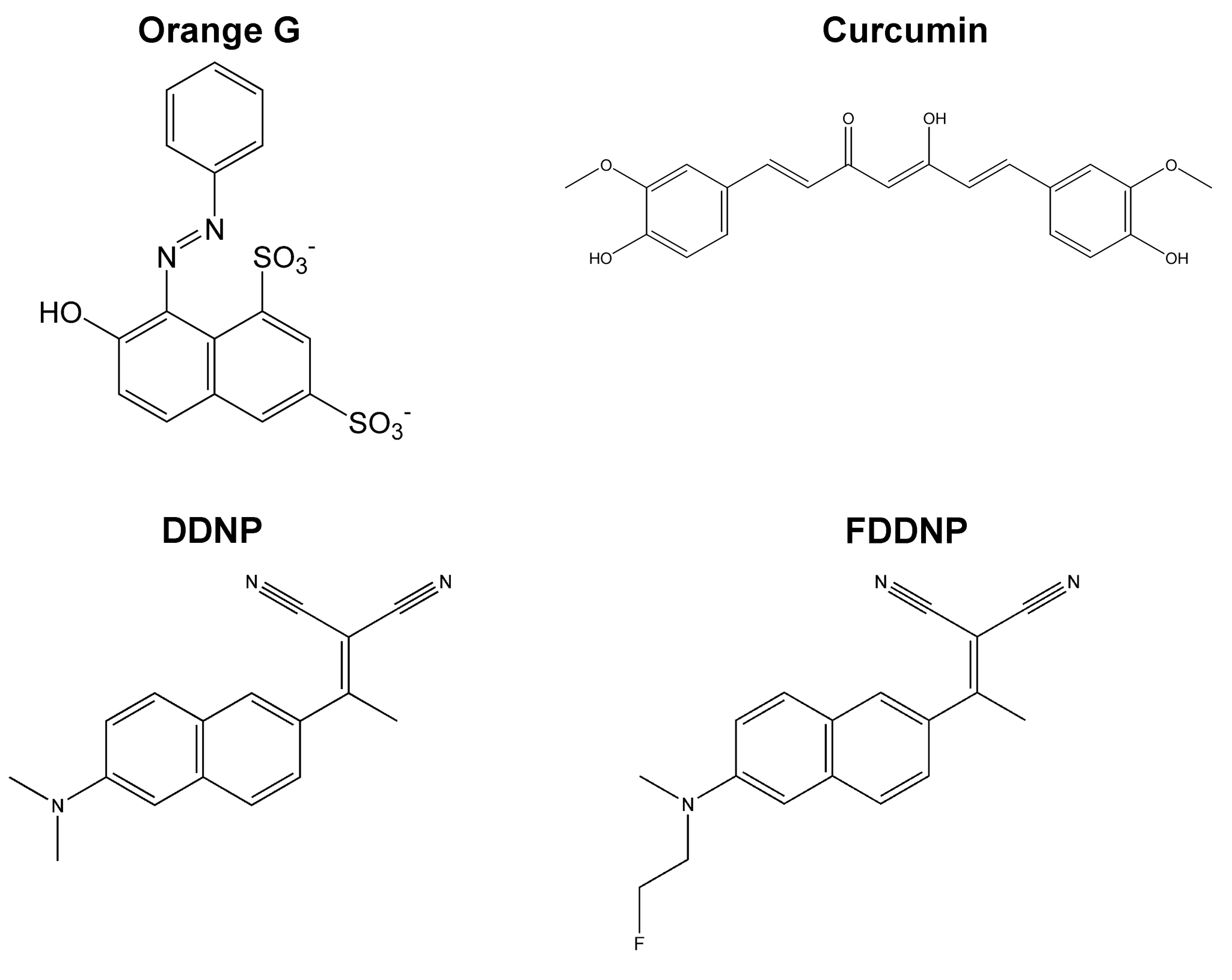

Supplement: Figure S1 — Chemical structures of the small molecule binders. (TIF) [file pbio.1001080.s001.tif]

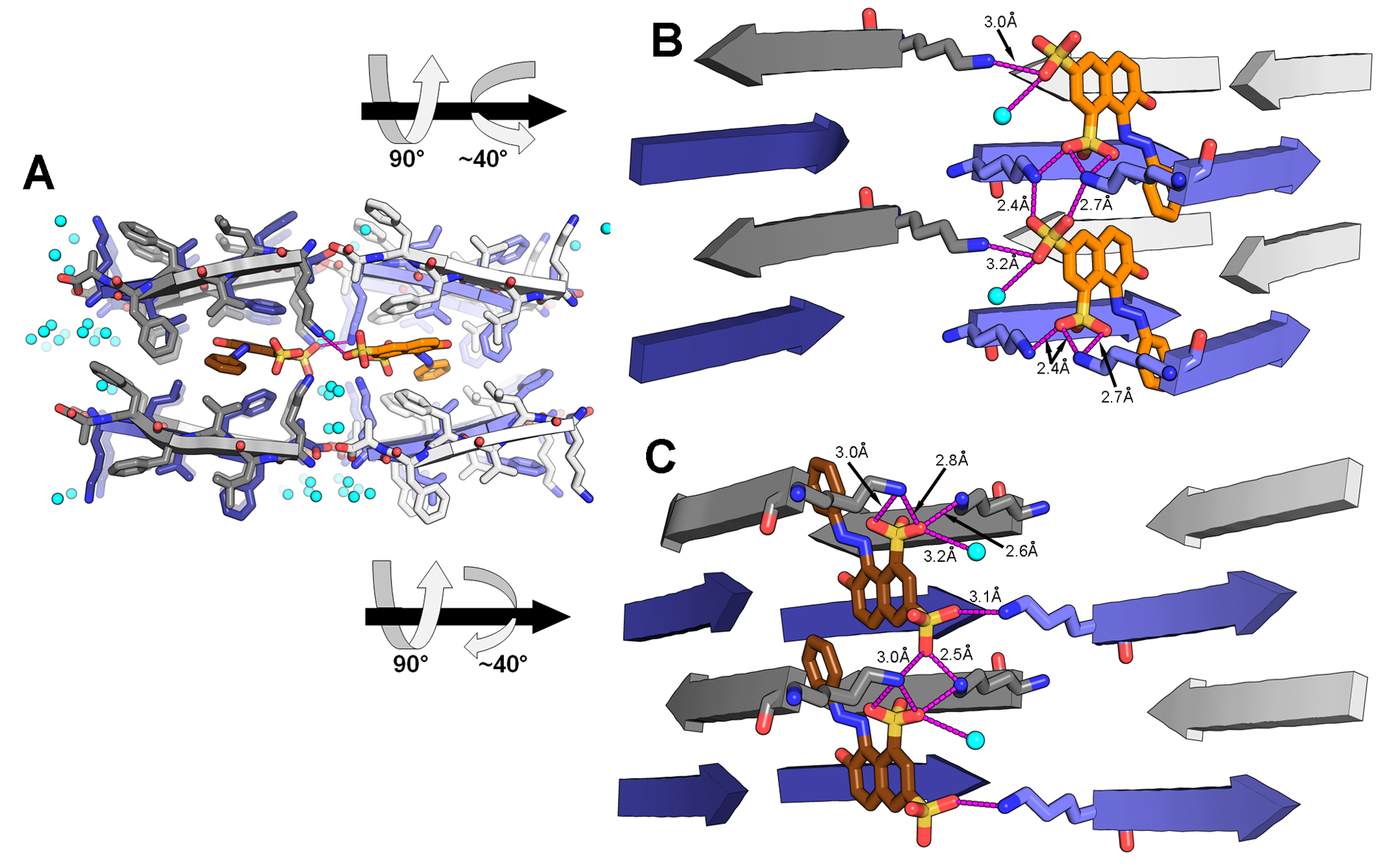

Supplement: Figure S2 — The crystal structure of the KLVFFA segment from Aβ complexed with orange-G shows extensive interactions between two orange-G molecules and the fiber. The KLVFFA segments are packed as pairs of β-sheets with orange-G bound internally to the steric zipper. The asymmetric unit of the crystal contains four peptide segments, two orange-G molecules, and 11 water molecules. Here, four layers of β-strands and two steric zippers are shown. KLVFFA and orange-G are shown as sticks with non-carbon atoms colored by atom type. The anti-parallel strands (cartoon arrows) are alternately colored white and blue, with the adjacent steric zipper colored in darker hues. The two orange-G molecules in the asymmetric unit, with carbons in orange and brown, display similar interactions with the fiber. In panel A, the view looks down the fiber axis. In panels B–C, the view is perpendicular to the fiber axis. The sulfonic acid groups of orange-G form salt links (pink lines) with five lysine residues, four protruding from facing β-sheets of the steric zipper and one from the adjacent zipper (only the latter is presented as pink lines in panel A). Only side chains of residues participating in salt links are shown. One of the sheets from the adjacent pair and one orange-G molecule are removed for clarity. (TIF) [file pbio.1001080.s002.tif]

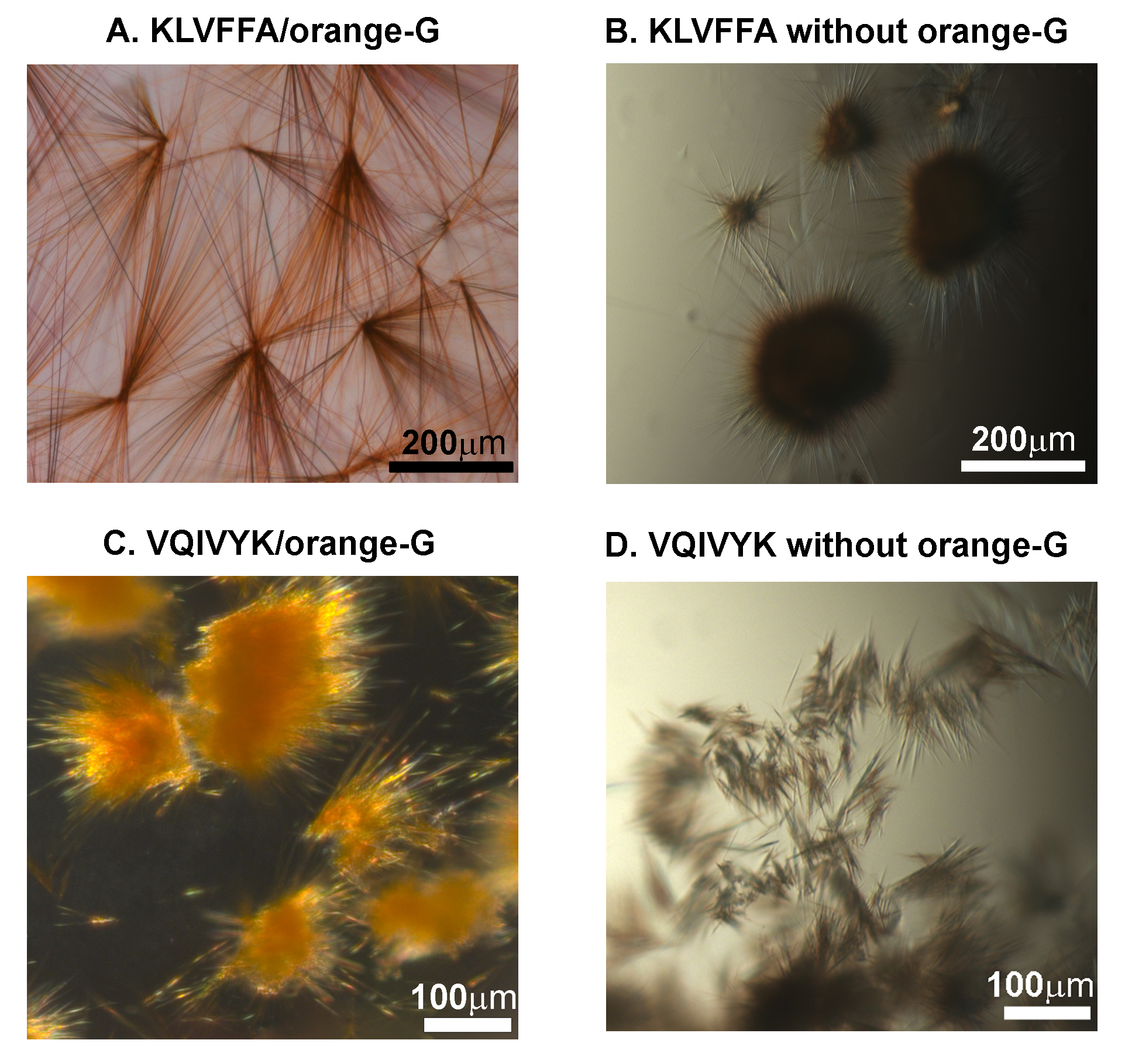

Supplement: Figure S3 — Crystals of the KLVFFA segment from Aβ and of the VQIVYK segment from the tau protein grown with and without orange-G. (A–B) Micro-crystals of the KLVFFA segment of Aβ grown under identical conditions (Methods) with (A) and without (B) orange-G. (C–D) Micro-crystals of the VQIVYK segment of the tau protein grown under identical conditions (Methods) with (C) and without (D) orange-G. (TIF) [file pbio.1001080.s003.tif]

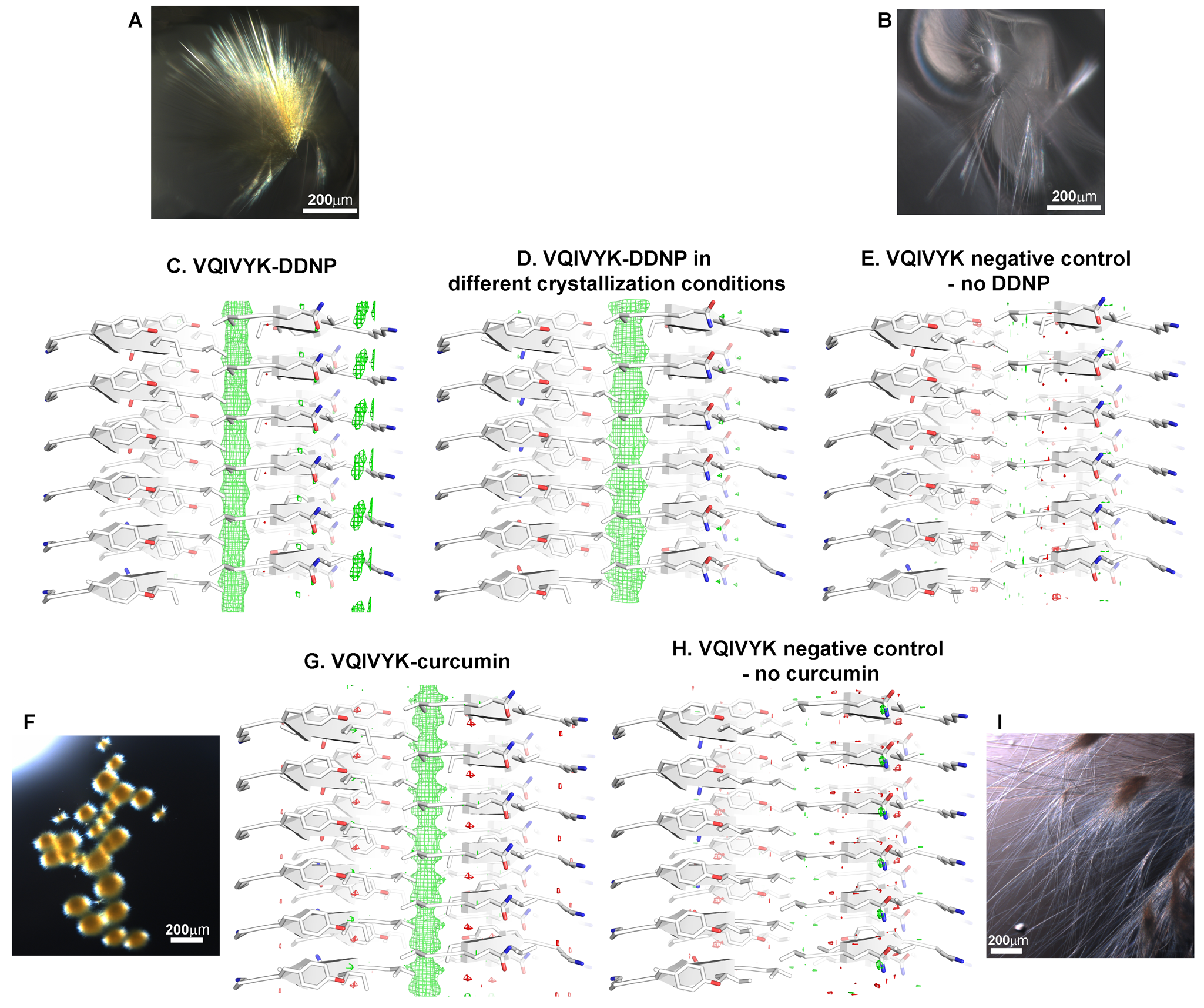

Supplement: Figure S4 — Crystal structures used as controls for the complexes of the VQIVYK segment from the tau protein with DDNP and curcumin. (A) Micro-crystals of VQIVYK co-crystallized with DDNP; the structure is shown in panel C. (B) Micro-crystals of VQIVYK crystallized under identical conditions to the crystals in panel A, lacking DDNP (Methods). The structure is shown in panel E. (D) The structure of VQIVYK co-crystallized with DDNP, grown under different crystallization conditions than the structure shown in panel C (Methods). (F) Micro-crystals of VQIVYK co-crystallized with curcumin; the structure is shown in panel G. (I) Micro-crystals of VQIVYK crystallized under identical conditions to the crystals in panel F, lacking curcumin (Methods). The structure is shown in panel H. In panels C–E and G–H, six layers of the VQIVYK fiber are depicted. The VQIVYK segment pack in parallel β-sheets (represented as cartoon arrows with white carbons). The view is perpendicular to the fiber axis, with β-strands running horizontally. Only the VQIVYK segment was modeled into the electron density. The difference electron density Fo-Fc map is shown as mesh (+3σ in green and −3σ in red), indicating missing atoms in the model. The crystals grown without the small molecule, either DDNP or curcumin (panels B and I, respectively), are colorless, whereas the co-crystals are colored (panels A and F, respectively). Moreover, the VQIVYKapo structures also lack the positive density (part of the structure that has not been modeled, green mesh) that we attribute to the presence of the small molecule (panels E and H versus panels C and G, respectively). Both structures of VQIVYK complexed with DDNP, grown under different crystallization conditions (panels C and D), show a similar, tube-like, positive electron density map, supporting the attribution of DDNP to this density. (TIF) [file pbio.1001080.s004.tif]

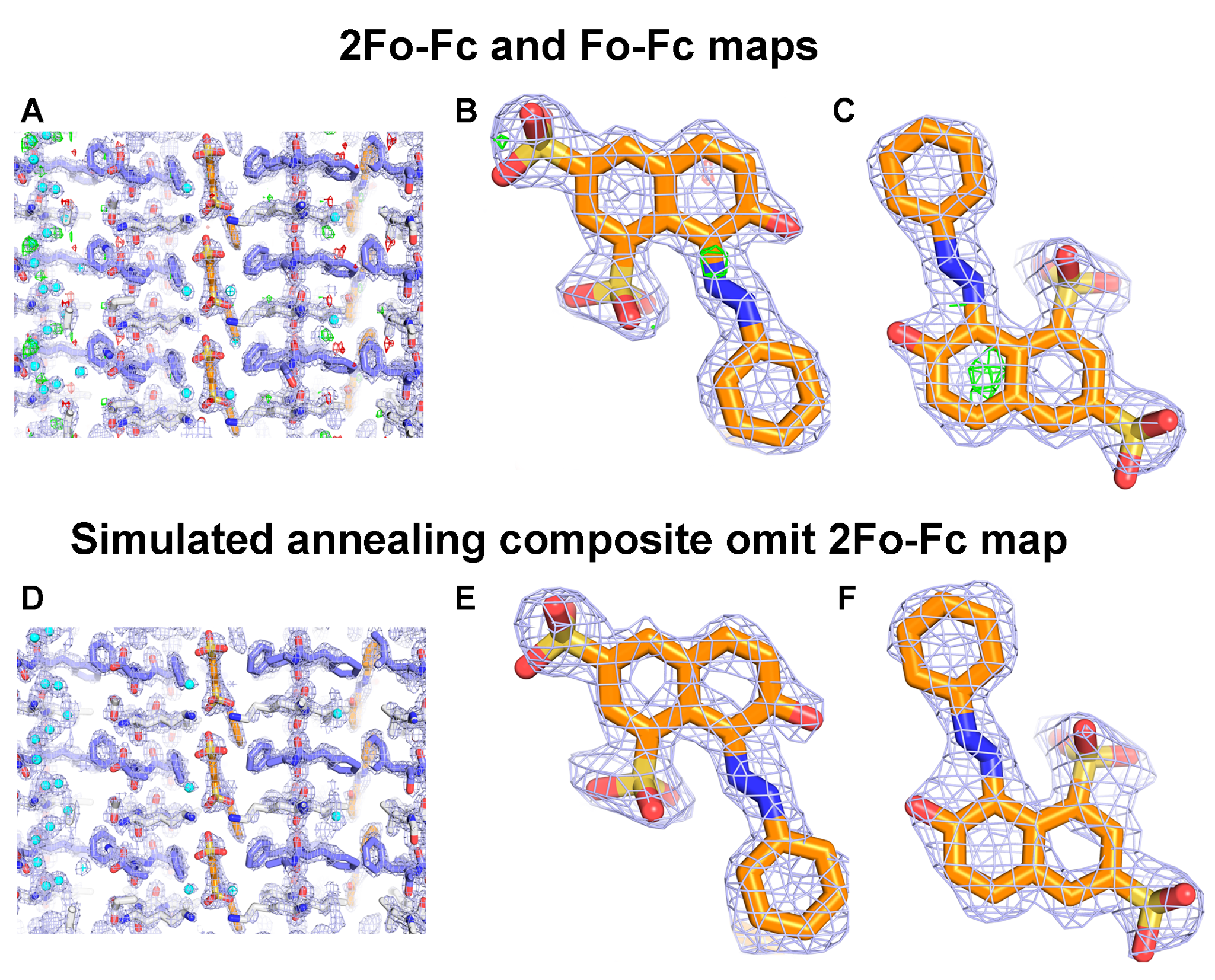

Supplement: Figure S5 — Electron density maps and simulated annealing composite omit maps of the KLVFFA segment from Aβ complexed with orange-G. The KLVFFA segments and orange-G molecules are shown as sticks with non-carbon atoms colored by atom type. The β-sheets are formed via stacks of anti-parallel strands, alternately colored with carbons in white and in blue. The carbons of the orange-G molecules are colored orange. Water molecules are shown as aqua spheres. The view here is perpendicular to the fiber axis. (A–C) The electron density 2Fo-Fc map is shown as grey mesh (1.3σ). The difference electron density Fo-Fc map is shown as mesh (+3σ in green and −3σ in red). (D–F) The simulated annealing composite omit 2Fo-Fc map (10% omitted) is shown as grey mesh (1.3σ). Panels B–C and D–E focus on the two orange-G molecules in the asymmetric unit. (TIF) [file pbio.1001080.s005.tif]

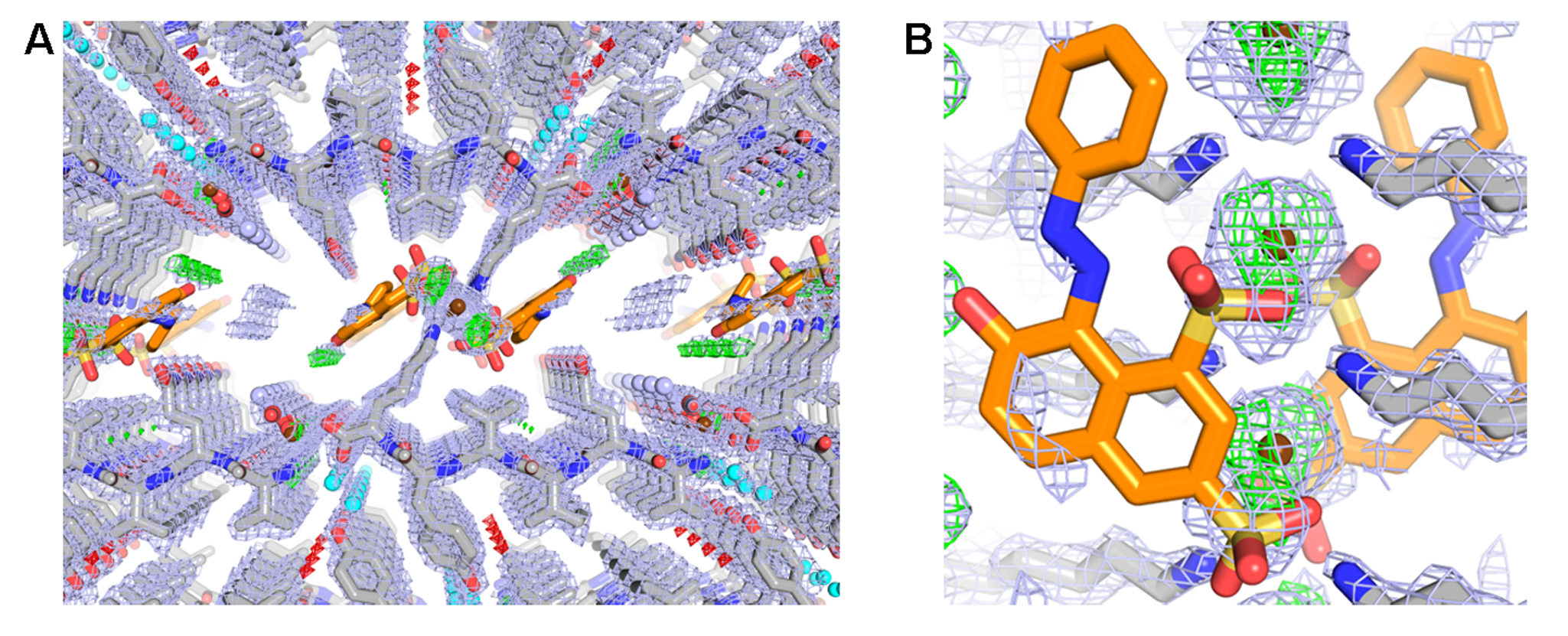

Supplement: Figure S6 — Electron density map of the VQIVYK segment from the tau protein complexed with orange-G. The VQIVYK segment and orange-G are shown as sticks with carbon atoms colored grey and orange, respectively, and non-carbon atoms colored by atom type. The view in panel A looks down the fiber axis. The view in panel B is perpendicular to the fiber axis and focuses on orange-G. The electron density 2Fo-Fc map is shown as grey mesh (1.3σ). The difference electron density Fo-Fc map is shown as mesh (+3σ in green and −3σ in red). (TIF) [file pbio.1001080.s006.tif]
