# Supplementary material for: Towards a Pharmacophore for Amyloid
Source: PLoS Biol. 2011 Jun 14;9(6):e1001080. doi: 10.1371/journal.pbio.1001080 (PMC3114762; doi:10.1371/journal.pbio.1001080)
Supplement: Table S1 — Screening for co-crystals from mixtures of amyloid-like segments with small molecules. (PDF) [file pbio.1001080.s007.pdf]

Towards a Pharmacophore for Amyloid

Meytal Landau, Michael R. Sawaya, Kym F. Faull, Arthur Laganowsky, Lin Jiang, Stuart A. Sievers, Jie Liu, Jorge R. Barrio and David Eisenberg

Supporting Table S1

Small Molecule	Amyloid-like segment	Co-crystallization result
Orange G	VQIVYK from tau	Structure of the complex was determined (Fig 3).
	KLVFFA (residues 16-21) from Aβ	Structure of the complex was determined (Fig. 1).
	KLVFFG (residues 16-21) – Flemish (A21G) mutation from Aβ	Crystals with x-ray diffraction too poor to determine structure.
	KLVFFAK (residues 16-22) – Italian (E22K) mutation from Aβ	No crystals.
	KLVFFAG (residues 16-22) - Artic (E22G) mutation from Aβ	Fibrous crystals.
	KLVFFAEN (residues 16-23) - Iowa (D23N) mutations from Aβ	Crystals with x-ray diffraction too poor to determine structure.
	KLVFFAENVG (residues 16-25) – Iowa (D23N) mutations from Aβ	No crystals.
	KLVFFAGNVGSNK (residues 16-28) - Artic (E22G) and Iowa (D23N) mutations from Aβ	No crystals.
	GDVGSNK (residues 22-28) – Artic (E22G) mutation from Aβ	No crystals.
	QDVGSNK (residues 22-28) - Dutch (E22Q) mutation from Aβ	No crystals.
	GGVVIA (residues 37-42) from Aβ	Crystals with x-ray diffraction too poor to determine structure.
	LVFFAEDVGSNKGAI IGLMVGGVV (residues 17-40) from Aβ	No crystals.
	LVFFAEDVGSNKGAI IGLMVGGVVIA (residues 17-42) from Aβ	Fibrous crystals.
	GVVEVD (residues 734-739) from Aβ A4 protein (APP)	Structure of orange-G alone.
	GDVIEV from α-crystalline	Crystals with x-ray diffraction too poor to determine structure.

	SSTNVG from amylin	All crystals formed (under various crystallization conditions) were colorless and not tested further.
	GNNQQNY from yeast prion protein Sup35	All crystals formed (under various crystallization conditions) were colorless and not tested further.
Curcumin	VQIVYK from tau	Structure of the complex was determined (Fig. 4).
	KLVFFA (residues 16-21) from Aβ	Crystals with x-ray diffraction too poor to determine structure.
	GGVVIA (residues 37-42) from Aβ	Structure of GGVVIA alone.
	GVVEVD (residues 734-739) from Aβ A4 protein (APP)	Crystals with x-ray diffraction too poor to determine structure.
	GDVIEV from α-crystalline	Crystals with x-ray diffraction too poor to determine structure.
	SSTNVG from amylin	Structure of SSTNVG alone.
Phenol Red	VQIVYK from tau	Crystals with x-ray diffraction too poor to determine structure.
	GGVVIA (residues 37-42) from Aβ	Structure of GGVVIA alone.
	SSTNVG from amylin	Crystals with x-ray diffraction too poor to determine structure.
	NFGAILSS (residues 22-29) from amylin	No crystals.
	SSNNFGAILSS (residues 19-29) from amylin	No crystals.
	SNNFGAILSS (residues 20-29) from amylin	Crystals with x-ray diffraction too poor to determine structure.
Thioflavin T	VQIVYK from tau	Structure of thioflavin T alone.
	KLVFFA (residues 16-21) from Aβ	Structure of KLVFFA alone.
	GGVVIA (residues 37-42) from Aβ	Crystals with x-ray diffraction too poor to determine structure.
	GVVEVD (residues 734-739) from Aβ A4 protein (APP)	Structure of GVVEVD alone in a unique anti-parallel packing.
	GDVIEV from α-crystalline	Crystals with x-ray diffraction too poor to determine structure.
Chicago sky blue 6B	VQIVYK from tau	Crystals with x-ray diffraction too poor to determine structure.
	GGVVIA (residues 37-42) from Aβ	Structure of GGVVIA alone.
Rhodamine B	VQIVYK from tau	Structure of VQIVYK alone.
	GGVVIA (residues 37-42) from Aβ	Crystals with x-ray diffraction too poor to determine structure.
Azure C	VQIVYK from tau	Crystals with x-ray diffraction

		too poor to determine structure.
	GGVVIA (residues 37-42) from Aβ	Crystals with x-ray diffraction too poor to determine structure.
Rolitetracycline	VQIVYK from tau	Crystals with x-ray diffraction too poor to determine structure.
	GGVVIA (residues 37-42) from Aβ	Crystals with x-ray diffraction too poor to determine structure.
Myristyltrimethyl -ammonium bromide	VQIVYK from tau	No crystals.
	GGVVIA (residues 37-42) from Aβ	Crystals with x-ray diffraction too poor to determine structure.
o-vanillin	VQIVYK from tau	Crystals with x-ray diffraction too poor to determine structure.
	GGVVIA (residues 37-42) from Aβ	Crystals with x-ray diffraction too poor to determine structure.
Juglone	VQIVYK from tau	Structure of VQIVYK alone.
	GGVVIA (residues 37-42) from Aβ	Structure of GGVVIA alone.
Hexadecyltrimeth -ylammonium bromide	VQIVYK from tau	Crystals with x-ray diffraction too poor to determine structure.
	GGVVIA (residues 37-42) from Aβ	Crystals with x-ray diffraction too poor to determine structure.
1,2- Naphthoquinone	VQIVYK from tau	Crystals with x-ray diffraction too poor to determine structure.
	GGVVIA (residues 37-42) from Aβ	Crystals with x-ray diffraction too poor to determine structure.
Lacmoid	VQIVYK from tau	No crystals.
	GGVVIA (residues 37-42) from Aβ	Fibrous crystals.
Perphenazine	VQIVYK from tau	Structure of VQIVYK alone.
Thioflavin S	VQIVYK from tau	Structure of VQIVYK alone.
Rifamycin SV sod. Salt	GGVVIA (residues 37-42) from Aβ	Crystals with x-ray diffraction too poor to determine structure.
	SSTNVG from amylin	Structure of SSTNVG alone.
Meclocycline sulfosalicylate salt	VQIVYK from tau	Crystals with x-ray diffraction too poor to determine structure.
Eosin Y	VQIVYK from tau	Fibrous crystals.
	GGVVIA (residues 37-42) from Aβ	Crystals with x-ray diffraction too poor to determine structure.
2,2′- Dihydroxybenzop -henone	GGVVIA (residues 37-42) from Aβ	Structure of GGVVIA alone.
Methylene Blue	VQIVYK from tau	Structure of Methylene Blue alone.
	GGVVIA (residues 37-42) from Aβ	Crystals with x-ray diffraction

		too poor to determine structure.
Benserazide hydrochloride	VQIVYK from tau	Fibrous crystals.
	GGVVIA (residues 37-42) from Aβ	Structure of GGVVIA alone.
2-Methoxy-4-methylphenol (Creosol)	VQIVYK from tau	Structure of VQIVYK alone.
	GGVVIA (residues 37-42) from Aβ	Crystals with x-ray diffraction too poor to determine structure.
R-(−)-Apomorphine hydrochloride hemihydrate	VQIVYK from tau	Crystals with x-ray diffraction too poor to determine structure.
	GGVVIA (residues 37-42) from Aβ	Structure of GGVVIA alone.
Dobutamine hydrochloride	VQIVYK from tau	Crystals with x-ray diffraction too poor to determine structure.
Neocuproine	VQIVYK from tau	Structure of VQIVYK alone.
(−)-Epigallocatechin gallate	VQIVYK from tau	No crystals.
	GGVVIA (residues 37-42) from Aβ	Structure of GGVVIA alone.
Epicatechin	VQIVYK from tau	No crystals.
PIB	VQIVYK from tau	Structure of VQIVYK alone.
	KLVFFA (residues 16-21) from Aβ	Crystals with x-ray diffraction too poor to determine structure.
DDNP	VQIVYK from tau	Structure of the complex was determined (Fig. 4).
	KLVFFA (residues 16-21) from Aβ	Crystals with x-ray diffraction too poor to determine structure.
	KLVFFG (residues 16-21) –Flemish (A21G) mutation from Aβ	Crystals with x-ray diffraction too poor to determine structure.
	KLVFFAK (residues 16-22) - Italian (E22K) mutation from Aβ	No crystals.
FDDNP	VQIVYK from tau	Crystals with x-ray diffraction too poor to determine structure.
	KLVFFA (residues 16-21) from Aβ	Structure of KLVFFA alone in a unique packing.
	LVFFAEDVGSNKGAI IGLMVGGVV (residues 17-40) from Aβ	Fibrous crystals with no x-ray diffraction.
	LVFFAEDVGSNKGAIIGLMVGGVVIA (residues 17-42) from Aβ	Crystals with x-ray diffraction too poor to determine structure.
CFDDNP	KLVFFG (residues 16-21) –Flemish (A21G) mutation from Aβ	Crystals with x-ray diffraction too poor to determine structure.
	KLVFFAK (residues 16-22) - Italian (E22K) mutation from Aβ	No crystals.
AZET	VQIVYK from tau	Structure of VQIVYK alone.
EB-I-68	KLVFFA (residues 16-21) from Aβ	Fibrous crystals.

Table S1. Screening for co-crystals from mixtures of amyloid-like segments with small molecules. We choose small molecules that were reported to affect fibrillation of different amyloid-forming proteins [1,2], including natural compounds [3], a thioflavin derivative: Pittsburgh compound B (PIB) [4], as well as a molecule that constitutes half of the curcumin molecule: (−)-2-Methoxy-4-methylphenol (Creosol). We also screened for complexes with biological marker that detect amyloid fibers *in vivo*, developed and synthesized by Jorge R. Barrio and co-workers [5-8].

We used 34 different small-molecules combined with different amyloid-like segments to generate 89 different mixtures. Several different molecular ratios (ranging between 1:1 and 1:10 small-molecule:segment) were tested (details not specified in the table) resulting in >100 different co-crystallization trials. Each mixture was screened for the formation of co-crystals with 768 different crystallization conditions. In many cases, crystals grown from various conditions were tested in x-ray diffraction experiments (details not specified in the table). Soaking experiments (adding the small molecule after growing crystals from the amyloid-like segment alone), performed for several of the different combinations, failed to show the presence of the small molecule.

From the 89 mixtures detailed in the Table, 4 structures of complexes were determined. 14 mixtures did not show formation of crystals in the conditions tested over several months. 47 mixtures resulted in fibrous or colorless crystals that were not tested, or crystals with x-ray diffraction too poor to allow structure determination. Crystals grown from 21 mixtures led to structure determination of the amyloid-like segment alone, while 3 revealed the presence of only the small molecule.

References

1. Necula M, Kayed R, Milton S, Glabe CG (2007) Small molecule inhibitors of aggregation indicate that amyloid beta oligomerization and fibrillization pathways are independent and distinct. J Biol Chem 282: 10311-10324.
2. Yang F, Lim GP, Begum AN, Ubeda OJ, Simmons MR, et al. (2005) Curcumin inhibits formation of amyloid beta oligomers and fibrils, binds plaques, and reduces amyloid in vivo. J Biol Chem 280: 5892-5901.
3. Choi YT, Jung CH, Lee SR, Bae JH, Baek WK, et al. (2001) The green tea polyphenol (-)-epigallocatechin gallate attenuates beta-amyloid-induced neurotoxicity in cultured hippocampal neurons. Life Sci 70: 603-614.
4. Mathis CA, Bacskai BJ, Kajdasz ST, McLellan ME, Frosch MP, et al. (2002) A lipophilic thioflavin-T derivative for positron emission tomography (PET) imaging of amyloid in brain. Bioorg Med Chem Let 12: 295-298.
5. Agdeppa ED, Kepe V, Liu J, Small GW, Huang SC, et al. (2003) 2-Dialkylamino-6-acylmalononitrile substituted naphthalenes (DDNP analogs): novel diagnostic and therapeutic tools in Alzheimer's disease. Mol Imaging Biol 5: 404-417.
6. Jacobson A, Petric A, Hogenkamp D, Sinur A, Barrio JR (1996) 1,1-Dicyano-2-[6-(dimethylamino)naphthalen-2-yl]propene (DDNP):A Solvent Polarity and Viscosity Sensitive Fluorophore for Fluorescence Microscopy. J Am Chem Soc 118: 5572-5579.
7. Shoghi-Jadid K, Small GW, Agdeppa ED, Kepe V, Ercoli LM, et al. (2002) Localization of neurofibrillary tangles and beta-amyloid plaques in the brains of living patients with Alzheimer disease. Am J Geriatr Psychiatry 10: 24-35.
8. Smid LM, Vovko TD, Popovic M, Petric A, Kepe V, et al. (2006) The 2,6-disubstituted naphthalene derivative FDDNP labeling reliably predicts Congo red birefringence of protein deposits in brain sections of selected human neurodegenerative diseases. Brain Pathol 16: 124-130.
