# Supplementary material for: Towards a Pharmacophore for Amyloid
Source: PLoS Biol. 2011 Jun 14;9(6):e1001080. doi: 10.1371/journal.pbio.1001080 (PMC3114762; doi:10.1371/journal.pbio.1001080)
Supplement: Table S2 — Data collection and refinement statistics (molecular replacement). (PDF) [file pbio.1001080.s008.pdf]

Towards a Pharmacophore for Amyloid

Meytal Landau, Michael R. Sawaya, Kym F. Faull, Arthur Laganowsky, Lin Jiang, Stuart A. Sievers, Jie Liu, Jorge R. Barrio and David Eisenberg

Supporting Table S2

	KLVFFA/orange-G	VQIVYK/orange-G
**PDB accession code**	3OVJ	3OVL
**Data Collection**		
**Beamline**	APS 24-ID-E	APS 24-ID-E
**Date**	2008/11/15 and 2009/11/13	2008/11/17
**Space group**	P 1	C 2
**Cell dimensions:**		
**a, b, c (Å)**	9.54, 26.01, 25.80	55.06, 4.83, 22.13
**α, β, γ (°)**	62.3, 88.6, 88.5	90.0, 103.0, 90.0
**Resolution range (Å)** [(*)]	26.01 - 1.8 (1.9 - 1.8)	55.06 - 1.8 (1.9 - 1.8)
**$R_{merge\ (square)}$ (%)**[(*)(a)]	18.9 (42.3)	17.9 (48.9)
**I/σI**[(*)]	4.38 (1.87)	3.55 (1.20)
**Completeness (%)** [(*) (b)]	91.5 (71.7)	87.6 (71.0)
**Redundancy**[(*)]	3.8 (1.7)	2.4 (1.4)
**Unique Reflections**	1870	587
**Refinement**		
**Resolution range (Å)** [(*)]	23.0 - 1.8 (2.0 - 1.8)	26.8 – 1.8 (2.0 - 1.8)
**Unique Reflections**	1692	525
**R_{work}(%)**[(*)(c)]**/ R_{free} (%)**[(*)(d) (e)]	20.5 (28.9) / 22.0 (36.6)	25.9 (37.7) / 26.0 (44.8)
**Completeness (%)** [(*)(b)]	92.9 (79.9)	88.0 (76.4)
**Observations/parameters ratio**	1.6	1.5
**Test set size [%], selection**	9.2, random	10.4, random

**Number of atoms in asymm. unit**	273	88
**Protein**	208	53
**Ligand**	54	33
**Water**	11	2
**Average B-factor (Å^2):**		
**Average B factor for main-chain atoms**	12.0	12.4
**Average B factor for side-chain atoms**	16.1	14.7
**Average B factor for ligands**	23.3	27.1
**Average B factor for water**	25.4	28.4
**R.m.s. deviations:**		
**r.m.s.d. bond length (Å)**	0.01	0.01
**r.m.s.d. bond angles (°)**	1.69	1.22
**R.m.s. B:**		
**R.m.s. B for main chain atoms**	1.0	0.9
**R.m.s. B for side-chain atoms**	2.7	2.9
**R.m.s. B for ligands**	7.0	2.0

Table S2. Data collection and refinement statistics (molecular replacement).

(*) Values in brackets are for the highest resolution shells.

(a) $Rmerge(square) = \sum (I - I(mean))^2 / \sum I^2$, where I is the observed intensity of the reflection HKL and the sum is taken over all reflections HKL.

(b) The incompleteness (<80%) of the highest resolution shell is likely due to the rapid decay of the peptide crystals that are naturally susceptible to radiation decay due to their small size.

(c) $Rwork = \sum \| F_o | - | F_c \| / \sum | F_o |$.

(d) R_{free} as defined by [1].

(e) The statistical significance of R_{free} is limited in the case of peptide structures, where the small size of the unit cell leads to a paucity of reflections. In these cases, the R_{free} statistic has proven to be a useful, but less sensitive tool compared with R_{free} statistics reported from macromolecular structure.

Reference

1. Brunger AT (1992) Free R value: a novel statistical quantity for assessing the accuracy of crystal structures. Nature 355: 472-475.
