# Supplementary material for: Towards a Pharmacophore for Amyloid
Source: PLoS Biol. 2011 Jun 14;9(6):e1001080. doi: 10.1371/journal.pbio.1001080 (PMC3114762; doi:10.1371/journal.pbio.1001080)
Supplement: Text S1 — (I) Using computational docking for structure determination. (II) Incommensurate structures. (PDF) [file pbio.1001080.s009.pdf]

Towards a Pharmacophore for Amyloid

Meytal Landau, Michael R. Sawaya, Kym F. Faull, Arthur Laganowsky, Lin Jiang, Stuart A. Sievers, Jie Liu, Jorge R. Barrio and David Eisenberg

SUPPORTING TEXT

I. Using computational docking for structure determination

In all four structures reported here, the electron density attributed to the small molecule was undifferentiated (to different extents), which hindered the determination of the structures in atomic detail. After assignment of the peptide segment into the electron density 2Fo-Fc map, the difference Fo-Fc map showed positive density that resembled a narrow and long tube running along the fiber (see e.g. in Figs. 6 and S4). This density indicated the presence of the small molecule, but was insufficiently detailed for atomic assignment. It is noteworthy that the small molecule constitutes a significant part of the asymmetric unit of the crystal in the complexes of small molecules with the peptide segments. For example, in the complex of KLVFFA with orange-G, the number of atoms of orange-G molecules constitutes ~20% of the total atoms in the asymmetric unit. Therefore, we anticipated that computational docking [1,2] (Methods) could be of help for the correct assignment of the small molecule atoms.

The generated docked structures were refined and evaluated based on their free-R value [3] (Methods). In the case of KLVFFA or VQIVYK with orange-G, the crystallographic refinement in the presence of the small molecule significantly decreased the free-R value (by 5% and 2%, respectively). In the case of VQIVYK with DDNP or curcumin, the refinement with the small molecule did not improve the free-R value and we concluded that the x-ray diffraction does not allow the determination of the position of the small molecule in atomic detail.

II. Incommensurate structures

In the three structures with VQIVYK complexed with orange-G, DDNP and curcumin, the lengths of the small molecules (DDNP ~12Å, curcumin ~19Å and orange-G ~9.5Å) span multiple unit cells of the fiber (4.8-4.9Å along the fiber axis; Figs 4 and 6); that is, the

dimensions of the small molecule and the fiber unit cell were incommensurate [4,5]. We postulated that the small molecule is drifting along the fiber axis, leading to disorder along this dimension.

Based on our structures we extrapolate that apolar compounds, such as DDNP and curcumin, bind to cylindrical cavities formed between pairs of β-sheets in amyloid structures. These cavities are frequently surrounded by hydrophobic and aromatic side chains [6,7], forming a binding motif for poly-aromatic compounds often reported to affect fibrillation [7-13]. Nevertheless, the binding is insufficiently specific, such that the molecules can be situated with different spacing along the fiber. Moreover, since the main constraint on binding is the width of the cylindrical cavity, the small molecule can not only drift along the fiber, but also rotate along its long dimension. In addition, one molecule can be flipped 180° relative to another molecule perpendicular to its long dimension. In the crystalline form, these degrees of freedom lead to the disorder seen in the electron density map attributed to the small molecules, precluding the determination of their position in atomic detail.

The binding of orange-G to the fiber-like structures is more specific than the binding of apolar compounds, via salt links between the negatively charged sulfonic acid groups of orange-G and the lysine side chains (Figs. 1 and 4). Congruently, the mass spectrometric analyses of the crystals showed that the molar abundance of orange-G in the crystals is high (~1:1 and ~1:10 stoichiometries with KLVFFA and VQIVYK, respectively) in comparison to the low molar abundance of curcumin and DDNP (~1:100 and ~1:400 stoichiometries with VQIVYK, respectively). Indeed, the structures of orange-G complexed with both KLVFFA and VQIVYK were more ordered, and the determination of the position of the orange-G in atomic detail was enabled using crystallographic refinements coupled with computational docking [1,2]. On the other hand, the low occupancy of DDNP and curcumin in the crystals, coupled with their possible drifting and rotation, corresponds to the disorder seen in the electron density map attributed to the small molecules, which prevented the determination of their position in atomic detail.

The high abundance of orange-G in the KLVFFA fiber corresponds to the detailed electron density for orange-G obtained following the computational docking (Fig. S5A-C). This electron density was validated via a simulated annealing composite omit map (Fig. S5D-F). In this

structure, the KLVFFA segment forms β-strands that are packed in an antiparallel orientation, associating to a unit cell dimension of 9.54Å along the fiber axis, which is sufficiently long to accommodate the orange-G (Fig. 1). Furthermore, we observed high complementarity between the chemical features of orange-G and the binding cavity on the KLVFFA fiber. The KLVFFA segment comprises a stretch of apolar side-chains preceded by a positively charged N-terminus. The apolar stretch, which includes aromatic side chains, attracts the aromatic rings of orange-G, while the lysine ammonium ions satisfy their charge by forming salt links to the negatively charged sulfate ions of orange-G (Figs. 1-2 and S2). In the complex with VQIVYK, the sulfate ions of orange-G again form a network of polar interactions (Fig. 4). However, the binding cavity of orange-G within the VQIVYK fibers is only 40% hydrophobic vs. the 80% hydrophobic cavity within the KLVFFA complex (Figs. 2 and 5). In the VQIVYK fibers, the aromatic rings of orange-G are packed against the hydrophobic side chains of Val4 and the carbon chain of Lys6, as well as against the polar side chain of Gln2 (Fig. 5). The differences in the binding cavities within the VQIVYK and KLVFFA structures may be responsible for the lower molecular abundance of orange-G in the VQIVYK co-crystals, to the incommensurate unit cell length, and to the resultant partial electron density observed for orange-G (Figs. S5-S6).

Supporting References

1. Davis IW, Baker D (2009) RosettaLigand docking with full ligand and receptor flexibility. J Mol Biol 385: 381-392.

2. Meiler J, Baker D (2006) ROSETTALIGAND: protein-small molecule docking with full side-chain flexibility. Proteins 65: 538-548.

3. Brunger AT (1992) Free R value: a novel statistical quantity for assessing the accuracy of crystal structures. Nature 355: 472-475.

4. de Wolff PM (1974) The Pseudo-Symmetry of Modulated Crystal Structures. Acta Crystallogr A 30: 777-785.

5. Hao Q, Liu Yw, Fan Hf (1987) Direct methods in superspace. I. Preliminary theory and test on the determination of incommensurate modulated structures. Acta Crystallogr A 43: 820-824.

6. Gazit E (2002) A possible role for pi-stacking in the self-assembly of amyloid fibrils. FASEB J 16: 77-83.

7. Porat Y, Abramowitz A, Gazit E (2006) Inhibition of amyloid fibril formation by polyphenols: structural similarity and aromatic interactions as a common inhibition mechanism. Chem Biol Drug Des 67: 27-37.

8. Porat Y, Mazor Y, Efrat S, Gazit E (2004) Inhibition of islet amyloid polypeptide fibril formation: a potential role for heteroaromatic interactions. Biochemistry 43: 14454-14462.

9. Ferrao-Gonzales AD, Robbs BK, Moreau VH, Ferreira A, Juliano L, et al. (2005) Controlling {beta}-Amyloid Oligomerization by the Use of Naphthalene Sulfonates: trapping low molecular weight oligomeric species. J Biol Chem 280: 34747-34754.

10. Necula M, Kayed R, Milton S, Glabe CG (2007) Small molecule inhibitors of aggregation indicate that amyloid beta oligomerization and fibrillization pathways are independent and distinct. J Biol Chem 282: 10311-10324.

11. Ono K, Yoshiike Y, Takashima A, Hasegawa K, Naiki H, et al. (2003) Potent anti-amyloidogenic and fibril-destabilizing effects of polyphenols in vitro: implications for the prevention and therapeutics of Alzheimer's disease. J Neurochem 87: 172-181.

12. Yang F, Lim GP, Begum AN, Ubeda OJ, Simmons MR, et al. (2005) Curcumin inhibits formation of amyloid beta oligomers and fibrils, binds plaques, and reduces amyloid in vivo. J Biol Chem 280: 5892-5901.

13. Cohen T, Frydman-Marom A, Rechter M, Gazit E (2006) Inhibition of amyloid fibril formation and cytotoxicity by hydroxyindole derivatives. Biochemistry 45: 4727-4735.
